# Approaches to the diagnosis and prevention of frailty

**DOI:** 10.1007/s40520-020-01559-3

**Published:** 2020-04-30

**Authors:** S. J. Woolford, O. Sohan, E. M. Dennison, C. Cooper, H. P. Patel

**Affiliations:** 1 Medicine for Older People, University Hospital Southampton, Mailpoint 63, G Level, West Wing, Tremona Road, Southampton, SO16 6YD UK; 2 MRC Lifecourse Epidemiology Unit, University of Southampton, Southampton, UK; 3 Academic Geriatric Medicine, University of Southampton, Southampton, UK; 4 NIHR Biomedical Research Centre, Southampton University of Southampton and University Hospital Southampton, Southampton, UK

**Keywords:** Frailty, Identifying frailty, Physical activity, Nutrition

## Abstract

An individual who is living with frailty has impairments in homeostasis across several body systems and is more vulnerable to stressors that may ultimately predispose them to negative health-related outcomes, disability and increased healthcare use. Approximately a quarter of individuals aged > 85 years are living with frailty and as such the identification of those who are frail is a public health priority. Given that the syndrome of frailty is defined by progressive and gradual loss of physiological reserves there is much scope to attempt to modify the trajectory of the frailty syndrome via physical activity and nutritional interventions. In this review we give an up to date account on the identification of frailty in clinical practice and offer insights into physical activity and nutritional strategies that may be beneficial to modify or reverse the frailty syndrome.

## Background

The syndrome of frailty is most associated with, but not an inevitable consequence of ageing and is characterised by a vulnerability to stressor events that can be both internal (e.g. infections and changes to medication) as well as external (e.g. changes in a person’s immediate environment or a breakdown in social care) [1, 2]. Frailty represents a considerable global healthcare burden, with an analysis of 21 cohorts of 61,500 community dwelling older adults, across mainly developed countries, estimating global frailty prevalence in those aged 80–84 years to be 15.7%, increasing to 26% in those aged > 85 years [3]. In the UK, NHS England estimate that 1.8 million people in the UK aged > 60 years are living with frailty, with diagnoses concentrated in those aged > 85.

Biological risk factors for the development of the frailty syndrome include age-related inflammatory processes, as well as common chronic diseases and their interactions with the environment. These factors lead to a physiological decline across multiple body systems, including skeletal muscle and bone, the cardiorespiratory system and the immune and endocrine systems. Other important contributors for developing frailty span non-physical domains. For example, cognitive frailty refers to cognitive decline in absence of dementia, social frailty refers to loneliness and the lack of robust social networks and psychological frailty refers to the psychological traits in an individual that may predispose them to a stressor event (e.g. a recent bereavement, low mood or a lack of motivation) [4, 5]. These conceptual constructs of the frailty syndrome highlight that managing a patient who is living with frailty requires a more holistic approach to managing the cause, or combination of causes, that has led to a diagnosis of frailty or a hospital admission for a given person [6].

**Fig. 1:**
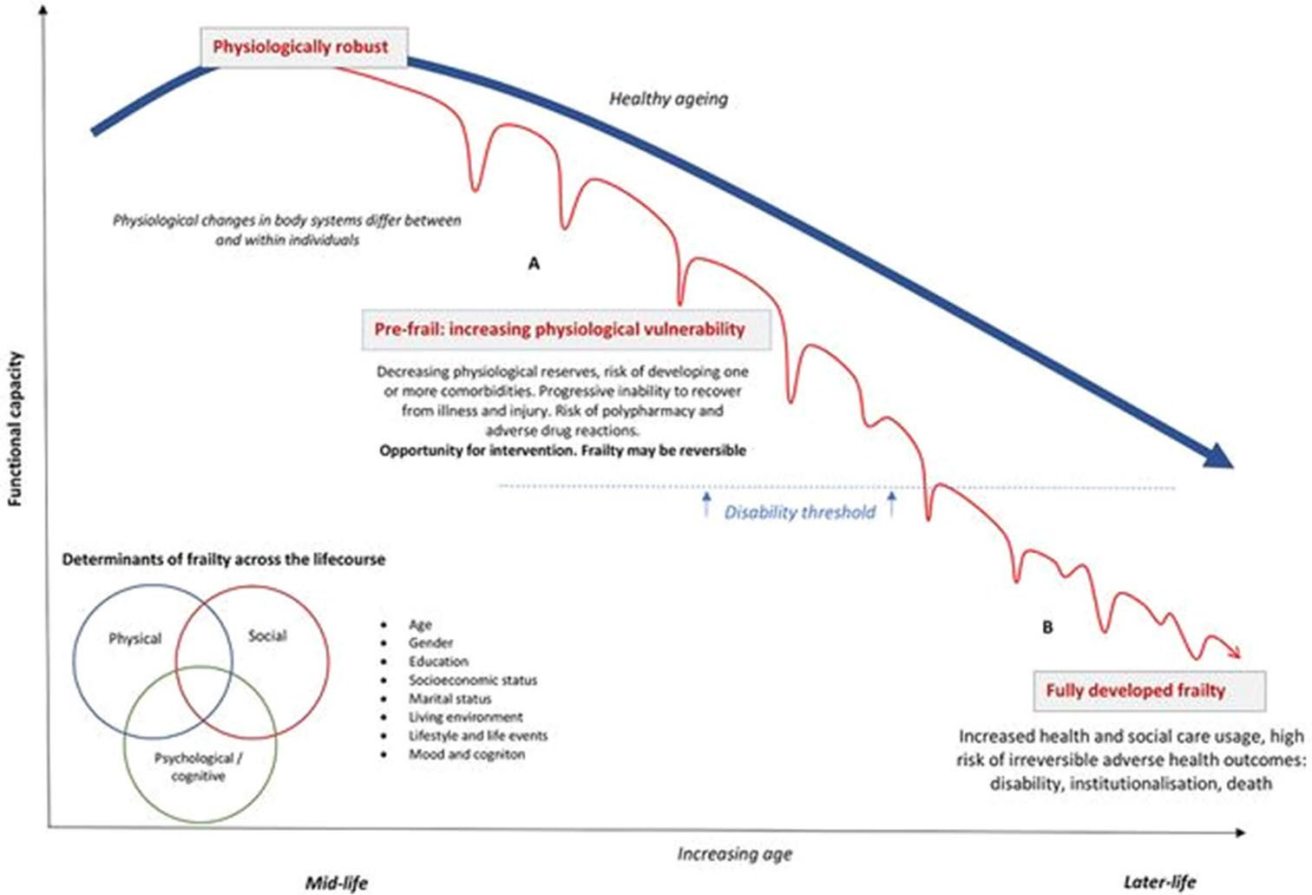
Determinants and development of frailty. The ideal healthy ageing paradigm, free of illness and physiological vulnerability, is represented by the thick line (blue). Frailty, represented by the thin line (red), develops as a continuum from a state of being physiologically robust and independent to being at risk of disability and dependency and, ultimately, to being hospitalised institutionalised or at risk of dying. In younger robust individuals (**a**) rapid recovery after an injury or illness that leads to reduced functional capacity is more likely. Later in the life course, cumulative physiological decline across multiple body systems leads to episodic functional, psychological or cognitive decompensation. At this stage, recovery after these stressor events takes longer as physiological and cognitive reserves are depleted. Eventually, a transition point in later life is crossed when the individual cannot compensate adequately and the ability to perform daily activity diminishes. This increases the likelihood of disability, healthcare service use or hospitalisation as a consequence of a relatively minor stresses or insults. Ultimately, the accumulation of these homeostatic insults results in further disability, healthcare dependency, recurrent hospitalisation, institutionalisation and risk of dying (**b**). [86] Adapted from Patel et al.

The accelerated loss of physiological reserves which characterises frailty can lead to wide range of common healthcare problems, such as a loss of strength, a reduction in mobility and subsequent falls, a reduced appetite and undernutrition, incontinence, sensory decline, and depression and anxiety. This accumulating dysregulation across multiple systems negatively impacts on previously normal homeostatic mechanisms, leading to a failure to return to a previous level of functioning after a stressor event and further acceleration of the development and progression of the frailty syndrome (Fig. 1). Ultimately, living with frailty predisposes an individual to increased healthcare dependency, including the use of multiple prescribed drugs, more frequent emergency hospital admissions and prolonged hospital stays, an accelerated transition to residential care, and ultimately, higher rates of mortality [7, 8].

In this article we aim to give an up to date account on the identification of frailty in clinical practice and offer insights into physical activity and nutritional strategies that may be beneficial to modify or reverse the frailty syndrome.

## Methods

A literature review was carried out utilising medical journal databases, including PubMed and the Cochrane Library. Search terms pertinent to this topic were used, such as “frailty”, “diagnosis”, “prevention”, “exercise”, “physical activity” and “nutrition”. Article titles and abstracts were then assessed for relevance and full-text screening performed if the title or abstract contained one or more search terms and the article itself was deemed relevant to this topic. Articles were also selected for further review from the wider literature based on the authors own expertise and knowledge of pre-existing work in this field. Only English language studies or studies translated into English were included.

## Identification of individuals living with frailty

Due to the disproportionate use of health and social care by individuals living with frailty, the routine identification and stratification of frailty across a variety of healthcare settings is crucial to enable the effective clinical management for this patient group. This can be facilitated through processes such as the comprehensive geriatric assessment (CGA). This is an evidence based multidimensional and interdisciplinary assessment of medical, psychological and functional capabilities aimed at developing an integrated plan for treatment and care of older persons. Furthermore, the process of CGA in itself is associated with favourable outcomes, such as reduced hospital length of stay and readmission rate, decreased mortality and improved cognition [9, 10]. The most established international models of frailty identification which can be assessed through such an assessment are the phenotype model, the cumulative deficit model and the clinical frailty scale (CFS), which are amongst a plethora of other frailty measurement systems each validated in local settings [11].

The phenotype model, developed by Fried et al., identifies frailty by the presence of at least three of five physical characteristics; weight loss, exhaustion, low energy expenditure, slow walking speed and low handgrip strength [12]. In contrast, the cumulative deficit model identifies frailty based on the accumulation of a range of symptoms, sensory deficits, clinical signs, diseases, disabilities and abnormal laboratory test results. Based on these metrics an index score is calculated, which is a function of the number of deficits present in an individual divided by the total number of deficits possible within the population sample [13]. The frailty index score which is generated, ranging from 0–1, is therefore a measure of accumulated vulnerability and indicates the likelihood of adverse outcomes [14]. In clinical practice, identification of frailty using the Canadian Study of Health and Ageing (CSHA) CFS is commonly used [13]. It is a simple tool based on a multidisciplinary assessment of physical, psychosocial, functional and environmental factors. This includes assessment of gait, balance, mobility, muscle weakness, osteoporosis risk, visual impairment, cognitive impairment and urinary incontinence. A frailty category is then assigned, ranging from 1 (fit) to 7 (severe frailty), with this system being designed to be easily used by the wider multidisciplinary team, such as occupational therapists, physiotherapists and social workers. The formulation of an individualised care plan for a given patient with frailty can then be informed by this frailty category.

In addition, an electronic frailty index (eFI) is now routinely available to be calculated on an automated basis from UK primary care system records. This is based on the cumulative deficit model and a total of 36 deficits spanning physical, social, psychological and cognitive domains are assessed [7]. The eFI identifies older people aged 65–95 with mild (eFI 0.12–0.24), moderate (0.24–0.36) or severe frailty (≥ 0.36) and has robust predictive capability for hospitalisation, institutionalisation and mortality.

The early identification of those who are frail can not only facilitate appropriate immediate clinical management for this patient cohort but can also enable the implementation of potential preventative or mitigating strategies by the multidisciplinary team, with exercise- and nutrition-based interventions being some of the most closely examined.

## Physical activity and the prevention of frailty

The degree to which somebody is physically active can directly contribute to the frailty syndrome in several ways. Firstly, physical inactivity can lead to a myriad of diverse chronic health issues, including cardiovascular disease, cerebrovascular disease, type two diabetes, depression and dementia. As mentioned previously, the effect of these conditions on physiological reserves can in turn result in the development or progression of the frailty syndrome. Secondly, a reduction in a person’s mobility or muscular strength are explicit diagnostic criteria for several frailty identification models, including the phenotype model, as mentioned previously. Lastly, a fall and the potential adverse sequalae following a fall, such as a bone fracture or a hospital admission, often represent an acute decompensation event for somebody with frailty, leading to a further loss in physiological reserve and a progression of the severity of their frailty. To this end, both North American and British guidelines advocate that older adults, particularly those at risk of falls, should carry out multi-component physical exercises that include both resistance- and balance-based activities [15, 16].

### Resistance-based exercise

Sarcopenia, which is the progressive and generalised loss of skeletal muscle mass, strength and function, is now recognised as a key component of the frailty syndrome [17]. One of the more traditional focuses of exercise-based interventions in older persons has been to improve muscle strength using resistance-based exercise programmes, reducing the development of sarcopenia and therefore also delaying the onset of frailty [18]. Resistance training has also been shown to decrease all-cause mortality and can improve functional performance, including posture, balance and endurance [19, 20]. Furthermore, in several studies where gait speed has been used as the primary outcome measure there is evidence to suggest that progressive resistance training with high intensities improves gait speed and hence decreases risks of falls, as well as morbidity and mortality [21–24].

In addition to preserving skeletal muscle, resistance exercise has also been shown to increase bone strength through repeated mechanical loading, thereby improving bone mineral density and reducing the development of osteoporosis [25]. A Cochrane systematic review carried out by Howe et al. examined 43 randomised controlled trials and found the most effective type of exercise for increasing neck of femur bone mineral density to be non-weight bearing high force exercise, such as progressive resistance strength training for the lower limbs [26]. More recently, resistance-based intervention programmes have focused on improving biomechanical imbalances that predispose to risk of falling, including abdominal trunk weakness, hip flexion and knee extension strength [27–29]. By reducing the risk of falls and subsequent fractures through improved bone mineral density acute decompensations and progression of the frailty syndrome may be mitigated.

### Balance- and functional-based exercise

A recent Cochrane systematic review of 108 randomised control trials performed by Sherrington et al. identified that exercise programmes which most effectively reduced falls primarily involved balance and functional exercises, as opposed to other physical exercise modalities [30]. In contrast, a recent meta-analysis carried out by Van Abbema et al. found that the addition of balance training or balance and endurance training to resistance training did not increase the positive effect of resistance training alone on gait speed. However, the authors identified that there was evidence to suggest that exercise with a rhythmic component, such as walking or dancing to music, may improve gait speed [31]. In addition to gait and balance training, there is increasing evidence that Tai Chi reduces the risk of falls in older adults [30, 32]. There are numerous components to Tai Chi, including mindfulness and active relaxation, but it is thought that the slow, rhythmic movements of Tai Chi improves balance control ability by focusing on the centre of gravity in constantly changing positions, as well as by strengthening lower limbs and increasing flexibility [33].

### Exercise and cognitive function

As mentioned previously, an appreciation of the psychosocial and cognitive aspects of frailty are important to fully understand how the syndrome develops and progresses. There have been significant advances in recent years in our understanding of how physical exercise may benefit cognitive function in older age. Aerobic exercise is thought to improve cognition via increased cerebral blood flow, thereby increasing oxygenation and providing energy for neurogenic and metabolic activity [34, 35]. Furthermore, there has been growing evidence that aerobic exercise produces changes in hippocampal structure and volume, resulting in the improved formation of new memories as well as improved spatial orientation [36, 37]. A recent meta-analysis by Firth et al. suggests that aerobic exercise retains volume in the left hippocampus and they concluded that more research is needed to further investigate the beneficial effects of aerobic exercise on other areas of the brain, including white matter regions and the basal ganglia [38]. More recently, the intensity of aerobic exercise and complexity of motor training tasks has been associated with global neuroplasticity, thereby enhancing overall cognitive function [39]. Furthermore, the additional benefits that rhythmic exercises and Tai Chi appear to generate above and beyond other balance-based exercises may be explained by these types of regimes improving cognitive function, as opposed to muscular or bone strength [31, 40].

Aside from its physical benefits, regular exercise has also been shown to be beneficial in the treatment of depression in older adults [41]. During exercise, endogenous opioid neuropeptides or endorphins are released from the pituitary gland, blocking neurotransmitters involved in the transmission of pain and creating a euphoric effect [42]. Regular exercise can also increase confidence, self-esteem and reinforce positive behaviours if the physical changes that occur create a sense of achievement. In older adults, regular group exercise has been shown to increase feelings of social connection within a community and the mutual social support can contribute to sustaining physical activity in the long term [43, 44]. As such, exercise-based interventions may be beneficial for reducing both social and psychological frailty.

As can be seen, encouraging regular exercise can promote healthy ageing and reduce the long-term health sequelae of frailty, thereby reducing its impact on healthcare systems. This has been supported by numerous studies positively associating regular exercise with reduced falls risk and improved balance, mobility, muscle strength and a reduction markers of frailty [45]. Whilst current evidence suggests that exercise in the older person should be a combination of aerobic and resistance-based exercise, more research is needed to reach a general consensus regarding the most effective exercise programme for an individual with frailty.

## Nutrition and the prevention of frailty

The role of nutrition in the prevention and progression of the frailty syndrome is becoming increasingly well understood, with several macronutrients and micronutrients having been identified as directly contributing to, or interacting with, frailty.

### Protein

Protein has received much attention in relation to the prevention of frailty due to its central role in muscle metabolism. Protein intake is the major contributor to muscle anabolism in older persons and contributes to the prevention of sarcopenia [46]. Sarcopenia, and the resulting impaired physical functioning, is a key contributor to the larger frailty syndrome due to its interaction with several widely accepted frailty criteria; namely low muscle strength, poor physical performance and reduced physical activity [1].

There is a wealth of observational data associating increased protein intake in older persons with improved physical functioning. The Health, Aging, and Body Composition (Health ABC) Study assessed protein intake amongst participants aged 70–79 years via questionnaire and assessed changes in body lean mass over 3 years [47]. They found that participants in the highest quintile of protein intake (on average 91 g/day) lost approximately 40% less lean mass compared to the lowest quintile of protein intake (on average 56.9 g/day). Other studies have gone onto directly associate raised levels of protein intake in older persons with reduced prevalence of frailty. One of the earliest notable studies investigating this topic was by Kobayashi et al. who investigated the associations between estimated protein intake in Japanese women aged > 65 years and frailty [48]. They found that total protein intake was inversely associated with prevalence of frailty when comparing the third, fourth and fifth quintiles of protein intake within their cohort to the first quintile. Several other studies have corroborated these findings, with a recent meta-analysis of four directly comparable cross-sectional studies concluding that protein intake was significantly negatively associated with frailty (OR 0.67, 95% CI 0.56–0.82, *p* = 0.0001) [49]. Interestingly, Kobayashi et al. also noted that the specific source of protein (i.e., whether the protein was derived from plant or animal) did not affect the prevalence of frailty [48]. Some consideration has also been given to the timing of protein consumption during the day. Bollwein et al. found that distribution of protein intake throughout the day, rather than total daily intake of protein, was associated with the prevalence of frailty, with frail participants consuming more protein at midday and less protein in the morning, and vice versa [50]. However, their findings have yet to be replicated by other studies.

Based on observational studies such as these it has been hypothesised that, for many older persons, protein intake may be inadequate and contributes to the development of frailty. Indeed, two USA-based studies noted that protein intake is less than the WHO Recommended Daily Allowances (RDA) of 0.8 g/kg/day in one third of women aged > 50 years and a quarter of men aged > 50 years [51, 52]. Furthermore, there is evidence to suggest that the current protein RDA is insufficient for older persons to maintain a stable muscle mass and maintaining protein intake at this level or below may result in a loss of skeletal muscle and subsequent development of sarcopenia [53, 54]. However, there has been much deliberation on what optimal and safe protein intake for older persons is. Increasing the protein content in older persons by a large amount may potentially lead to a damaging increase in metabolic workload for the kidneys due to the increased amounts of protein catabolism [55]. Additionally, older frail patients tend to struggle to consume additional oral supplements, even with help from specially trained staff [56] and poor concordance with nutritional supplements has been described due to a range of gastrointestinal side effects, such as diarrhoea [57]. It should also be noted that protein supplementation in older persons can reduce appetite and induce early satiety, reducing the intake of other crucial dietary components and worsening aged-related weight loss [58].

Several intervention studies investigating a range of potential hyperproteic dietary regimes have produced inconsistent results. A major meta-analysis by Milne et al. in 2006 of 55 trials of protein and energy supplementation in older persons found that dietary supplementation reduced overall mortality and improved outcomes for those admitted to hospital [57]. However, these results were only borderline significant and largely limited to patients with pre-existing malnutrition within the hospital population, as opposed to community population. Based on this current evidence base, there has been expert consensus opinion in recent years recommending a protein intake of ≥ 1.0–1.2 g/kg/day for healthy older people, and of 1.2–2.0 g/kg/day of protein intake for those who are malnourished or at risk of developing further comorbidities, i.e. those who are frail or pre-frail [59, 60]. However, these is little evidence yet as to whether this regime translates into an improvement in patient outcomes. One recent 12 week double-blind, placebo-controlled randomised controlled trial has assessed the effects of protein intake of 0.8, 1.2, and 1.5 g/kg/day, which is within the range of this new recommended protein intake, on muscle mass, strength, and physical function in older persons who are pre-frail and those who are frail and at risk of malnutrition [61]. They found that the intervention was safe and that amongst those who received 1.5 g/kg/day there was an improvement in appendicular lean mass and gait speed. However, there was no significant improvement in physical function, muscle strength or the recorded frailty index. Further evidence is clearly needed supporting the efficacy of this dietary regime before definitive public health advice can be given regarding protein intake and prevention of frailty amongst older persons.

### Calorie intake

Low energy intake is very common amongst older persons, with calorific intake progressively declining by approximately 25% between the fourth and seventh decades of life [62]. Furthermore, reduced energy intake is even more prevalent in older persons who are residents in long-term healthcare facilities, such as care homes [63]. This reduced calorific intake has been associated with reduced muscle mass, reduced physical performance, weight loss and increased disability [62, 64], which are all factors closely associated with the frailty syndrome. To sufficiently meet resting energy expenditure in older persons it has been calculated that, on average, 25 kcal/kg/day is needed, which is increased to approximately 32.5 kcal/kg/day when accounting for normal physical activity [65]. Several studies have found that calorie intake lower than this level is associated with increased prevalence of frailty in older persons. The InCHIANTI study found that daily energy intake of less than 21 kcal/kg was associated with frailty [66]. Furthermore, in a 10 year longitudinal observational study, women who had daily intake of less than 25 kcal/kg had a three-fold increased risk of dying or becoming frail [67] and the recent Rotterdam study found that in their cohort for every 100 kcal increase the prevalence of frailty was 5% lower [68]. However, there is little interventional evidence to support directly supplementing the diet of older persons calorifically. The previously mentioned meta-analysis by Milne et al. found no significant positive associations between calorific supplementation, when combined with protein supplementation, and morbidity for the community older person population, but did find a modest improvement in mortality and morbidity amongst those in hospital [57]. However, Kim et al. later found that energy supplementation of 400 kcal/day, alongside protein supplementation of 25 g/day, did improve physical performance and significantly slowed the rate of decline in gait speed and the timed “up-and-go” test [69].

### Vitamin D

Vitamin D has remained a focus of nutritional supplementation in the context of frailty due to multiple large observational studies associating 25 hydroxy-vitamin D (25(OH)D) deficiency with poorer physical performance, disability and frailty. The Third National Health and Nutrition survey (NHANES III) found that 25(OH)D deficiency (defined as < 15 ng/ml) was associated with a 3.7 times increased risk of developing frailty [70]. Similar results were produced in the InCHIANTI study, which found that pre-frail subjects with 25(OH)D levels < 20 ng/ml were 8.9% more likely to die or to become frail than pre-frail subjects with 25(OH)D levels > 20 ng/ml [71]. Furthermore, a recent meta-analysis of seven other observational studies found that, when comparing the lowest recorded level of 25(OH)D to the highest recorded level, older persons were 27% more likely to be frail [72].

Despite these promising data, evidence to support the efficacy of vitamin D supplementation to prevent frailty is inconsistent. A meta-analysis of 13 randomised controlled trials by Muir et al. found that supplementation of 800–1000 international units of 25(OH)D in adults aged > 60 years was associated with improvements to strength and balance [73]. However, another meta-analysis of 17 randomised controlled trials by Stockton et al. found no associations between 25(OH)D supplementation and grip or proximal lower limb strength [74]. Interestingly, they did find a positive association between vitamin D supplementation in those who were 25(OH)D deficient (defined as < 25 nmol/l) and hip strength. This finding is supported by a study by Bischoff-Ferrari et al. in which post-menopausal vitamin D deficient women with an average 25(OH)D level of 13.2 ng/ml were found to have a 2.8 fold increase in lower extremity function after daily supplementation with 20 μg of 25(OH)D [75]. Therefore, targeted vitamin D supplementation in those who are already vitamin D deficient may result in improved physical functioning, and therefore reduced prevalence of frailty, in later life. However, there has yet to be any studies investigating the effects of vitamin D supplementation on the development of frailty specifically.

### Mediterranean diet

Due to the complex synergistic relationships that individual dietary components display which each other effort has been made to investigate how more generalised dietary patterns may relate to frailty. One of the most widely evaluated dietary patterns is the Mediterranean diet. There are varying definitions of the Mediterranean diet, but most include higher daily intake of vegetables, fruits, cereals, olive oil and fish with comparatively lower intakes of red meat, processed meat, sweets and dairy compared to other diets [76]. Whilst the benefits of the Mediterranean diet have been extensively documented in relation to cardiovascular disease, cancer and neurodegenerative disorders [77], several observational studies have specifically examined the associations between adherence to the Mediterranean diet and frailty. In a cross-sectional study Bollwein et al. found that in German adults aged > 75 years greater compliance with the Mediterranean diet was associated with a significantly reduced prevalence of frailty [78]. Similar findings were observed using the prospective cohort from the Italian InCHIANTI study [79]. In community-dwelling adults aged > 65 years after 6-year follow-up closer adherence to the Mediterranean diet was associated with a lower risk of developing frailty. These findings have also been replicated outside of the Western setting, with Chan et al. describing a similar reduction in frailty prevalence in those who followed a Mediterranean diet in rural China [80]. Finally, a recent meta-analysis of four comparable observational studies found that greater adherence to the Mediterranean diet was significantly associated with a reduced incidence risk of frailty (OR 0.44, 95% CI 0.31–0.64, *p* < 0.001, for a Mediterranean diet score of 6–9) [81]. Considering that increased comorbidity status is thought to contribute to the development of frailty [82], it is perhaps unsurprising that the Mediterranean diet, which has wide-ranging benefits in relation to many disease processes, is so clearly preventative for the development of frailty.

## Conclusions and future research

Older individuals living with frailty use considerable resources in the last 12 months of life. Identifying individuals who are vulnerable, pre-frail or are living with frailty should be an essential part of assessment of older people as this aids health and social case resource allocation, service planning and can inform the implementation of potential preventative strategies by the multidisciplinary team.

Whilst several individual dietary or physical activity interventions have clear potential benefits for those who are pre-frail or frail the existing evidence base is too inconsistent to recommend the optimal mode of a single physical activity or a single dietary regime for the prevention of frailty [83, 84]. However, there is emerging evidence for the synergistic benefits for combined activity and nutritional interventions for the older person living with frailty. One study of particular interest is SPRINTT (Sarcopenia and Physical Frailty in Older People: Multi-Component Treatment Strategies), a large, multi-centre randomised controlled trial with a key goal being to evaluate the effectiveness of a multi-component intervention in preventing mobility disability in the older person. Participants were randomly assigned to either a multi-component intervention group which incorporated a physical activity intervention, a nutritional assessment and dietary intervention and a health technology intervention or a placebo lifestyle education programme that met twice a month. The SPRINTT study is due to end on the 30th June 2019, after which optimal treatment and management options for the older person living with frailty and sarcopenia may be identified and whether the frailty trajectory can indeed be modified by such a combined physical activity and nutritional intervention approach [85].
